# Wolfram Syndrome-2, a Cause of Severe Gastrointestinal Bleeding: A Case Series and a Literature Review

**DOI:** 10.1097/PG9.0000000000000339

**Published:** 2023-07-17

**Authors:** Rania Ateya, Thomas Ciecierega, Muttaz Abusamra, Motee Abuawwad, Abdulsalam Abu-Libdeh, Mutaz Sultan

**Affiliations:** * Al-Quds University, East Jerusalem, Palestine; † New York Presbyterian - Weill Cornell Medical College, New York, NY; ‡ Makassed Hospital – Al-Quds University, Abu-Dies, East Jerusalem, Palestine.

**Keywords:** *CISD2* gene, DIDMOAD, optic atrophy, gastrointestinal ulcers, diabetes insipidus

## Abstract

**Background:**

There are very few reports of Wolfram syndrome-2 (WFS2) in the literature, and understanding of involvement of the gastrointestinal (GI) tract in the syndrome is limited. Objectives: This study aims to describe the clinical presentations of a large number of WFS2 patients with specific focus on their GI manifestations.

**Methods:**

This is a retrospective case series study. Patients who were homozygous for the *CISD2* gene mutation were identified through the genetic department of Al-Makassed hospital. Their medical records were reviewed, and biometric data have been obtained. The data were collected and arranged on a data sheet, and descriptive analysis was done using SPSS.

**Results:**

Thirteen patients from 9 families were identified; diabetes mellitus was present in 6 of them, optic atrophy in 5, diabetes insipidus (DI) in 5, and deafness in 2. All of the patients had GI manifestations with abnormal findings on upper endoscopy. Dysmorphic facial features and abnormal findings on brain MRI were present in 3 of our patients. The GI manifestations including GI bleeding and severe ulcerations were the first to appear in 9 of them, while anemia in the remaining 4.

**Conclusion:**

This is the largest study to date describing patients with WFS2. This study’s evidence shows the prominent presence of GI involvement, and the severe findings on endoscopy, including duodenal, gastric, and esophageal ulcerations and strictures. Unlike in the Jordanian report, some of the patients in our report also have DI.

**What Is Known**

- Wolfram syndrome (WFS)-2 is a consequence of a mutation in *CISD2* gene, characterized mainly by diabetes mellitus and optic atrophy.
- WFS2 is a newly described variant from WFS1. Deafness is reported in both of them, while diabetes insipidus is associated with WFS1.
- Severe gastrointestinal (GI) bleeding episodes are reported in WFS2 but not WFS1.

**What Is New**

- WFS2 patients have severe GI ulceration and strictures on endoscopy.
- All our cases presented with anemia or GI complaints; some continue to have only these manifestations without developing any other component of WFS2.

## INTRODUCTION

Wolfram syndrome (WFS), which was first described in 4 siblings by Dr. Wolfram in 1938 (1,2), is a progressive neurodegenerative disorder, defined by the combination of diabetes mellitus (DM) and optic atrophy (OA) as the main criteria for diagnosis (3–6).

The frequent association of diabetes insipidus (DI) and deafness in WFS patients led to the term DIDMOAD (DI, DM, OA, and deafness) (7). The syndrome usually presents as a timed sequence of abnormalities, commonly starting from juvenile onset DM (first decade), followed by OA, central DI and deafness (second decade) (7), renal tract abnormalities (third decade) (2), and neurological manifestations in the fourth decade of life (3,5).

WFS has a very wide range of manifestations and a variable clinical picture among cases reported in the literature. The DM and the OA are the main criteria for the diagnosis of WFS. The syndrome should be suspected in any patient with these abnormalities (2–4,6).

## ETIOLOGY

Two causative genes were identified in WFS. *WFS1* gene is localized on chromosome 4p16.1, encoding for Wolframin protein, which causes WFS1 (1,7). The *CISD2* gene is on chromosome 4q22, and encodes for endoplasmic reticulum intermembrane small (ERIS) protein, which was recently identified to be responsible for WFS2 (7,8). Both are inherited in an autosomal recessive pattern (6,9).

## ASSOCIATED MANIFESTATIONS

Several associated abnormalities in WFS1 were reported in the literature. These include neurological, psychiatric, renal, and GI manifestations (4,7).

The reported associated neurological manifestations include horizontal nystagmus, seizures, mental retardation, truncal ataxia, lower limb areflexia, startle myoclonus, central apneas, cerebellar dysarthria, autonomic neuropathy, loss of taste or smell, hemiparesis, extensor plantares, and reduced peripheral sensation (4,5).

Associated psychiatric manifestations have also been reported, including episodes of severe depression, psychosis, organic brain syndrome (2), as well as, compulsive verbal and physical aggression. Carriers for the WFS mutation were found to be 8 times more susceptible than the noncarriers to be hospitalized due to psychiatric illness and suicide (4).

Additionally, associated renal manifestations included dilated renal outflow tract with urinary incontinence, increased frequency and recurrent infections, abnormal urodynamic study results (5). Atonic bladder, low-capacity or low-compliance bladder, detrusor external sphincteric dyssynergia, and emptying problems were also reported (3,4).

Associated GI manifestations in the form of GI dysmotility including chronic constipation or diarrhea were reported in a UK nationwide study of the syndrome (5)

Nevertheless, reported GI-associated manifestations in WFS2 are still limited, EL-Shanti et al reported severe profound peptic ulcer disease with GI bleeding in 11 out of 16 patients from 4 families. Additionally, bleeding tendency with abnormal coagulation tests results were also reported (10).

## PATHOPHYSIOLOGY

*WFS1* gene was found to be abundant in pancreatic beta cells, brain, heart, and muscle cells and to a lesser extent in the liver and kidneys. The pathophysiology is still not fully understood (6). However, current evidence suggests that the mutated *WFS1* gene is coding an endoplasmic reticulum (ER) transmembrane protein (1), which is when defective leads to loss of homeostasis and apoptosis in the cells (8).

WFS2 is suggested to be caused by a mutation in *CISD2* gene on chromosome 4q22. Similarly to *WFS1* gene, it encodes for an ER transmembrane protein (3,11). A single missense mutation in this gene, which results in a premature stop codon, was identified. The mutated gene is thought to cause defect in the calcium homeostasis leading to atrophy and degeneration to the pancreatic and brain cells just like it has been suggested in WFS1 (8).

Furthermore, CSID2 was suggested to be responsible for mammalian lifespan control. As it is also localized in the mitochondrial outer membrane (8). Chen et al, in their study on mice with knocked-out *CISD2* genes, reported progressive mitochondrial dysfunction and autophagic cell death leading to accelerated aging process, in the nervous system, muscles, and pancreatic cells, all which match OA and DM seen in WFS2 patients (12).

## WFS1 VERSUS WFS2

WFS2 is a newly reported variant of WFS. It was described by Ajlouni et al in 3 Jordanian families, where patients had DM, OA, and deafness but no DI. It is characterized by the associated urinary tract dilatation, impaired renal function, hypogonadism, severe GI ulcers, and bleeding tendency (6,9).

Reported patients showed a prominent GI bleeding tendency compared with the previous reported cases in the literature. Ninety-one percent (10/11) of the patients in the study who underwent upper GI endoscopy had abnormal findings, including severe duodenal ulcers and active gastritis causing serious episodes of an upper GI bleeding. Additionally, excessive bleeding was identified while taking the biopsies during the endoscopy. The coagulation studies showed abnormalities in 79% (11/14) of the tested patients in the form of platelets aggregation dysfunction and collagen abnormality (9).

After the Jordanian report of the 3 consanguineous families with WFS2 (9), only 2 reports describing 3 cases were published in the literature, 2 Italian siblings, (13) and one Caucasian girl (14). Thus, the reports and studies on WFS2 in the literature are still very limited, and detailed evidence on GI manifestations in WFS2 is still missing.

This study aims to provide the clinical picture of a relatively large number of WFS2 patients, with a focus on the associated GI manifestations. This will allow physicians to better identify syndrome’s comorbidities and improve patients’ clinical, medical, and endoscopic management.

## METHODS

This study is a retrospective case review. Genetically confirmed WFS2 patients were identified at the genetic department at Al-Makassed Hospital (a tertiary medical care center in Jerusalem). An ethical committee approval was obtained from Al-Quds University. All previous patients’ reports at Al-Makassed hospital were reviewed in detail and summarized on a previously prepared sheet that included their biometric data. The descriptive data analysis was performed via SPSS.

The variables were organized into 5 categories as follows: (1) patient demographic information including age, gender, and parents’ consanguinity; (2) the syndrome’s disease presentation, including types of clinical disease, age of onset, complains, and complications; (3) specific focus on GI manifestations, including complaints, endoscopy results, and complications; (4) Others, including screening for comorbidities and reviewing kidney and liver function test, coagulation profile, history of anemia, and previous need for packed red blood cells (PRBCs) or intravenous iron; and (5) a review of any additional laboratory tests or imaging investigations.

## RESULTS

**FIGURE 1.**
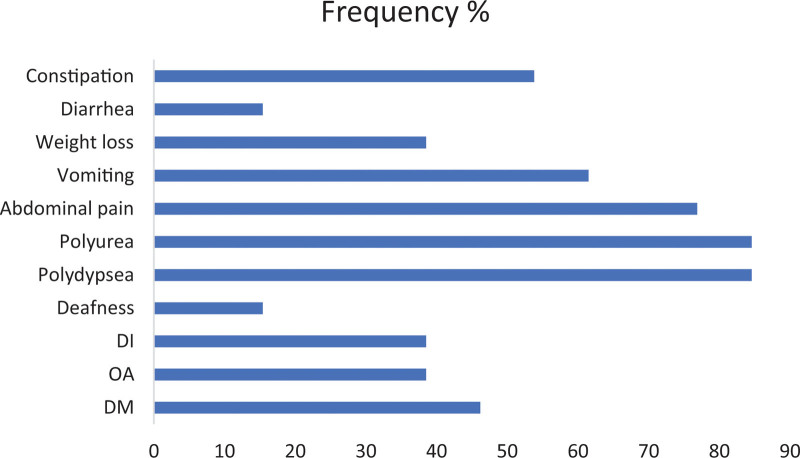
Describes the frequency in percentile of the 4 main components of the syndrome and the common manifestations among our patients.

**FIGURE 2.**
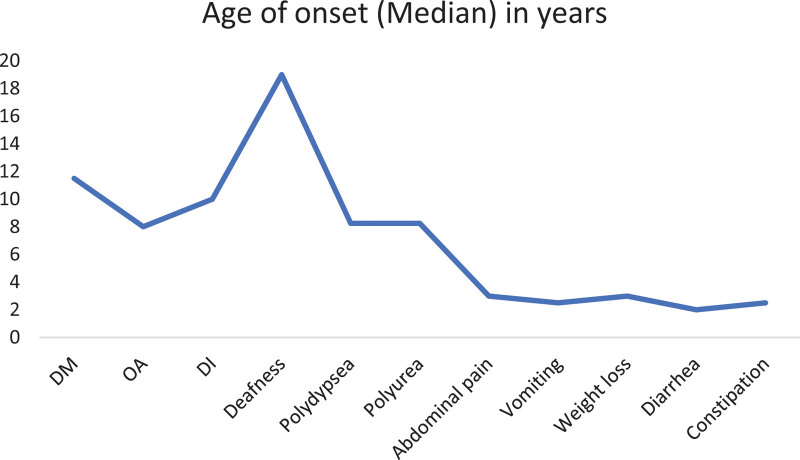
Shows the age of onset (represented by the median) of each of the main components of the syndrome and the associated manifestations. DI = diabetes insipidus; DM = diabetes mellitus; OA = optic atrophy.

Figures 1 and 2 summarize the frequency and age of onset of the 4 main components of the syndrome and the associated manifestations among the patients. A total of 13 patients from 9 families were identified via the genetic testing. All had confirmed WFS2 (homologous for the mutation E37Q in exon 2 of the *CISD2* gene, which was detected before in WFS2 as a disease-causing mutation (15)). Eight patients were males (61.5%) and 5 patients were females (38.5%). The age at last GI follow-up visit ranged from 2.5 to 22 years old with a mean of 10.1 years old. Parents were first cousins in 9 cases (69.2%), distant relatives in 2 cases (15.4%), and no consanguinity was identified in 2 cases (15.4%).

Six cases (46.2%) had DM, 5 cases (38.5%) had OA, and 5 cases (38.5%) had DI. Hearing impairment was identified in only 2 patients (15.4%) who were the oldest (17 and 22 years old) among our subjects.

In patients with DM (n = 6), the age of onset was reported in 4 of them and it ranged from 8.5 to 12 years old, with a mean of 10.9 and median of 11.5 years old. In OA groups (n = 5), the age was reported in 3 patients: 3, 8, 9.5 years old. In DI patients (n = 5), the age was reported in 4 of them: 9, 10, 10, 14 years old with a mean of 10.75 years old. Polydipsia and polyuria were present in 84.6% of the patients. Age was reported in 10 of the 11 patients, ranging from 2.5 to 12 years old with a mean of 7.3 and median of 8.25 years old. Four of the 11 patients had neither DM nor DI.

None of the patients were reported to have a diabetic ketoacidosis event, although 3 patients (23.1%) reported hypoglycemic episodes. Highest Hga1c readings in the diabetic patients ranged from 6.4% to 11.5% with one missing value. The mean was 7.9%.

A prominent finding in our study was the involvement of the GI system, and the GI manifestations were the first manifestations to appear in 9 cases (69.2%); all before the age of 5 years old. The remainder 4 patients had anemia as their first manifestation.

Interestingly, 4 patients, aged 2.5, 5, 5.5, and 10.5 years old, respectively, had none of the main components of WFS at last follow-up visit. However, they all had the GI manifestations as their main morbidity.

History of GI bleeding was evident in all our patients and all underwent esophagogastroduodenoscopy with abnormalities found in all the cases. The findings on upper endoscopy included the following: duodenal ulcer (n = 11; 84.6%), duodenal stricture (n = 8; 61.5%), esophageal ulcers (n = 8; 61.5%), esophageal stricture (n = 1; 7.7%), gastric ulcer (n = 4; 30.8%), and gastritis (n = 3; 23.1%). GI surgeries to bypass obstruction were indicated in 5 patients (38.5%).

All patients had history of anemia, with mean hemoglobin level at presentation of 6 gm\dL. PRBCs were needed in 11 out of the 13 patients (84.6%) and the number of units used was reported in 4 of them to be 2, 2, 3, 4 units. Intravenous iron infusion was reported to be needed in 4 patients (30.8%).

All patients had GI manifestations. The earliest was at the age of 1.5 years old, which was abdominal pain. Abdominal pain was the most commonly reported GI symptom, presenting in 76.9% of the patients with the mean and median age of onset of 5.3 and 3 years old, respectively. Vomiting was reported in 8 patients (61.5%) with age at onset ranging from 1 to 11 years old, mean of 3.5 and median of 2.5 years old. Constipation was reported in 7 patients (53.8%) with age documented in 5 of them, ranging between 1.5 and 17 years old, with a mean of 5.3 and median of 2.5 years old. Other symptoms included weight loss, documented in 5 patients (38.5%) and diarrhea, documented in 2 patients (15.4%).

Two patients were diagnosed with associated celiac disease based on the positive serology and duodenal biopsy changes. Two other patients had positive *Helicobacter pylori (H pylori*) test.

MRI was performed in 3 siblings among our patients and showed abnormal findings including nonenhancing microadenoma and nonfunctioning pituitary microadenoma; however, their significance in relation to the DI remained unclear. Focal area of encephalomalacia in the high right parietal lobe with few tiny brain foci in the right cerebellar hemisphere were most likely due to an old minor ischemia.

Dysmorphic facial features were recognized in 3 of our patients, including downslanting eyes, hypertelorism, large ears, prominent philtrum, and gingival hypertrophy.

Three of our patients had reported kidneys abnormalities on ultrasound, including single functional kidney in 2 of them and unilateral kidney hypertrophy in the third patient. However, their kidney function tests (creatinine and BUN) were within normal limits.

Coagulation profile was reviewed including Prothrombin Time, Activated Partial Thromboplastin Time, International Normalized Ratio, and platelets count. All our patients showed normal values throughout their medical care at our center. However, platelet function test was not performed in any of the patients because of unavailability of the test. In addition, liver markers (Alanine Transferase, Aspartate Transferase, and Alkaline Phosphatase) were reviewed, and were found to be normal in all of our patients.

## DISCUSSION

The patients in our study showed the prominent and strong presence of GI manifestations in setting of WFS2. GI manifestations and anemia were the first abnormalities to appear in all of our patients; this was also seen in the 3 reported cases in the literature after the Jordanian study.

A distinctive finding in our study is that some genetically confirmed WFS2 patients have none of the DIDMOAD features, and the GI manifestations were their main morbidity.

All patients had abnormal findings on the upper endoscopy. Findings included duodenal ulcers and strictures, gastritis, gastric ulcers, esophageal ulcers, and strictures, with the latter findings being uncommon. The ulcers were severe in most of our patients with some requiring diversion surgeries. We tested the fasted gastrin levels in some of our patients in an attempt to explain the GI ulcerations that were found on endoscopy, all of which were normal. In addition, only 2 of the patients were positive for *H pylori*, which does not explain the severity and extend of the ulcers. Although the underlying pathophysiology of the GI ulcers is not well understood, it could be a result of the mitochondrial and ER dysfunction that result from the *CISD2* gene mutation, which lead to degeneration and autophagic cell death. However, this explanation need to be further investigated (8).

GI ulcers, together with platelet dysfunction that has been described in the Jordanian study (9), collectively are more likely to result in the sever GI bleeding and anemia that our patients presented with.

In contrast to the Jordanian study (9) and the reported Caucasian case (14), which reported the absence of DI in WFS2 patients, some of our patients had DI and improved once on treatment. This observation was also reported by the Italian reported cases, which could not corroborate the absence of DI as it was present in one of the 2 cases (13).

Our study is similar to the Italian case report in its finding, while the Caucasian girl showed findings relatively similar to that reported in the Jordanian study.

Neither dysmorphic features nor abnormal MRI findings were reported before in WFS2 patients.

Although psychological evaluation is important to assess the presence of associated psychological manifestations and the impact of the disorder on an individual’s mental health, the young age of our patients and the scarce availability of pediatric psychological service were the main reasons for not undergoing psychological evaluation.

Parents consanguinity has been reported in most of our affected patients assessed by verbal medical history. Therefore, a founder effect is a possible explanation for the high frequency of a rare genetic disorder in a specific population and small geographic area. However, determining the presence of a founder effect would require detailed genetic analysis on a large scale of population in the affected area.

## CONCLUSION

Our study shows that WFS2 patients can suffer from severe GI ulcerations as a presenting manifestation, and they in fact might be causing an underlying anemia. DI, unlike in other reported cases, was present in only some of our patients. Some of our patients showed dysmorphic facial features and some had abnormal brain MRI findings, which were not reported in the literature before. Our study describes the clinical picture in a relatively large number of WFS2 patients in the literature so far.

### Limitations

Since this is a retrospective study that depends on multiple physicians’ reports, some data could have been incomplete because of the variability in documentation among the physicians. Furthermore, most of the patients were followed in many health care centers (including other hospitals, outpatient clinics, and laboratories), which might have limited investigators access to available test results.

## ACKNOWLEDGMENTS

The authors thank the resident physicians at the pediatric department of Al-Makssed Hospital, with special mention of Dr. Bisan Ayman, and the genetic department staff for their cooperation and Dr. Ibrahim Ghannam for statistical consultation.
